# Natural Antimicrobials: A Reservoir to Contrast *Listeria monocytogenes*

**DOI:** 10.3390/microorganisms11102568

**Published:** 2023-10-15

**Authors:** Annalisa Ricci, Camilla Lazzi, Valentina Bernini

**Affiliations:** 1 Department of Food and Drug, University of Parma, Viale delle Scienze, 49/A, 43124 Parma, Italy; camilla.lazzi@unipr.it (C.L.); valentina.bernini@unipr.it (V.B.); 2 SITEIA.PARMA, Viale delle Scienze, Tecnopolo, Padiglione 33, 43124 Parma, Italy

**Keywords:** anti-listeria activity, fermentation, plant extracts, plant-based antimicrobials, natural resources

## Abstract

Natural environments possess a reservoir of compounds exerting antimicrobial activity that are forms of defence for some organisms against others. Recently, they have become more and more attractive in the food sector due to the increasing demand for natural compounds that have the capacity to protect food from pathogenic microorganisms. Among foodborne pathogens, *Listeria monocytogenes* can contaminate food during production, distribution, or storage, and its presence is especially detected in fresh, raw food and ready-to-eat products. The interest in this microorganism is related to listeriosis, a severe disease with a high mortality rate that can occur after its ingestion. Starting from this premise, the present review aims to investigate plant extract and fermented plant matrices, as well as the compounds or mixtures of compounds produced during microbial fermentation processes that have anti-listeria activity.

## 1. Introduction

Natural environments possess a great number of different compounds deriving from all living organisms. Among this huge reservoir, some of these compounds possess activity against microorganisms such as bacteria. These activities are forms of defence for some organisms against others [1]. For example, vegetal organisms produce antimicrobial compounds to protect themselves against phytopathogens, and animals do the same to counteract the attack of pathogenic microorganisms [2]. However, plants and animals are not the only organisms that are able to produce antimicrobial compounds. The same microorganisms can synthesize and release compounds that inhibit the growth of other microorganisms by ensuring themselves nutrients, environmental space, etc. [1]. This great availability of compounds can be exploited to find new and effective antimicrobials that can be employed in various fields. Among them, the food sector seems, in recent years, to be very interested in finding efficient preservatives of natural origin due to the pressure of consumers being more attracted to clean-label foods, which limits the use of chemical preservatives that are perceived as unhealthy [3,4].

Simultaneously, food safety is a topic of great actuality because foodborne diseases are often related to the ingestion of microbially contaminated food. A wide number of microbial species are recognised to be involved in foodborne illnesses; however, among them, some were considered to be more dangerous for human and animal safety.

*Listeria monocytogenes*, a ubiquitous microorganism, can cause a series of diseases of severe intensity. Despite the low number of listeriosis cases, it has a high death rate and, for this reason, is considered a major public health issue [5]. Listeriosis can occur as an invasive or non-invasive infection. The most common symptoms in healthy people are headache, stomach ache, fever, diarrhoea, and vomiting, but it can also lead to intense symptoms in people in risk groups (for example, pregnant women, infants, old people, etc.) such as meningitis, sepsis, miscarriages, and even death [6]. Overall, listeriosis continues to be one of the foodborne infections with the highest number of fatal cases in the European Union, particularly among elderly people [7].

Due to its ubiquity, *L. monocytogenes* can be found in food products along the food production chain, in primary production, manufacturing, and distribution, leading to 1482 confirmed human infections in the EU in 2021. These cases resulted in 923 hospitalizations and 196 deaths [7]. Different foods, especially ready-to-eat products, can be contaminated by *L. monocytogenes.* In 2021 in the EU, the highest occurrences were observed for fish and fishery products (3.5–5.4%), meat products from bovine or pig origin (2.7–3.9%), fruits and vegetables (2.5%), and hard cheeses from raw or low-heat-treated sheep milk (4.6%) [7].

The greater adaptability of this foodborne pathogen to different conditions creates great concern about this microorganism and its presence in foodstuffs. Indeed, it can tolerate saline environments and multiply in a wide range of pH values (4.4–9.4), as well as being able to resist water activity higher than 0.92 [6]. Its optimum growth temperature ranges between 30 and 37 °C; however, it can survive and multiply at low temperatures (0–4 °C) [8]. Although, *L. monocytogenes* does not manifest an extraordinary resistance to high temperatures, even if its resistance is also related to the food matrices that it invades [9,10]. To ensure the safety of food and inhibit the presence of *L. monocytogenes*, whilst at the same time matching the requirements and preferences of consumers, new and effective anti-listeria solutions must be investigated.

Starting from these premises, the present review aims to investigate and enclose the knowledge on those compounds present in nature that have anti-listeria activities. Various plant extracts possessing anti-listeria activity have been reported in recent years. However, more recently, the fermentation process has become attractive for the increasing number of works showing the contribution of this process to antimicrobial activity. To hold all this information, the last ten years papers on plant extracts and the fermented parts of plants are included in this review.

## 2. Plant-Based Extracts: Anti-Listeria Activity

Due to the great potential of antimicrobial compounds that are included in plants and, consequently, in their derived extracts, there are a lot of works focusing their attention on this topic. More than 70 papers (especially published in the last five/six years) studied the anti-listeria activity of more than 100 different plants and/or parts of them (Table 1). The activity of all these extracts was studied in vitro with different techniques; however, most of the presented papers also investigated the anti-listeria activity in food matrices. All these matrices possess high heterogeneity and can potentially be sources of compounds that exert an adverse effect against *L. monocytogenes*. The anti-listeria activity of the leaves of different plants was studied, as well as the flowers, seeds, roots, and the entire plant (Table 1). Bulbs, fruits, and by-products were studied as well, but to a lesser extent (Table 1).

Plant extracts include a great variety of compounds, but not all possess anti-listeria activity. The compounds that can be found in an extract depend on different factors: the composition of the extracted plant, the solvent, and the employed technique of extraction [62]. Water, ethanol, and methanol are the solvents that have been most employed, followed by some other solvents (Table 1). However, the antimicrobial activity possessed by an extract can be the result of the extraction of a single compound or the activity of more compounds, as well as on their dose. In addition, the inhibitory effect against *L. monocytogenes* also depends on the interspecies variability of the same microorganism, as its ability to be resistant to compounds which it has previously come into contact with often depends on the source of isolation.

### Compounds Exerting Anti-Listeria Activity in Plant Extracts

The antimicrobial compounds in plant extracts are secondary metabolites with a defensive role for the plant, secreted by epidermal plant cells [2]. Among them, polyphenols, lignans, alkaloids, glycosides, saponins, tannins, and antimicrobial peptides can be found [2,86].

Polyphenols, one of the most numerous and important secondary metabolites, are ubiquitous in plants. They are usually involved in the defence of plants (against oxidizing agents, ultraviolet radiation, and phytopathogens) [2]. A lot of studies have reported the antimicrobial activity of polyphenols, and some of them are included in the work of Zamuz et al. [6]. Different phenolic compounds exert anti-listeria activity, such as hydroxycinnamic acids, anthocyanidins, flavan-3-ols, flavonols [82], oleuropein, verbascoside, luteolin-7-*O*-glucoside, luteolin-4-*O*-glucoside [74], anthocyanins, flavonols, phenolic acids [63], tannins, flavonoids [76], and quercetin [87]. The OH functional groups are related to the antibacterial activity of many phenolics; indeed, in phenolic compounds, the OH groups interact with the cell membrane via hydrogen bonding [6,88]. This antimicrobial activity can be ascribed to the modification of the cell membrane permeability, the disruption of the cytoplasmatic membrane, and the modification of pH as a consequence of an improper flow of H^+^ and K^+^, as well as dysregulating the proton motive force. However, intracellular damage can also be due to the presence of phenolic compounds, due to the formation of hydrogen bonding among phenolic compounds and enzymes or the inhibition of energy production [6,88].

In addition, lignans can be part of plants and are widespread in pteridophytes, gymnosperms, and angiosperms, being one of the earliest forms of defence [2].

Alkaloid compounds can also be found in plants, especially dicotyledons, acting as antimicrobial components. Some authors have reported the activity or the possible activity of these compounds against *L. monocytogenes* [40,79]. Kim et al. (2013) demonstrated that the anti-listeria activity observed in a *Corydalis turtschaninovii* rhizome extract was related to the different alkaloids that were in the extract. This study also analysed the mechanism of action of these compounds against *Listeria*. Microbial cells treated with dehydrocorydaline showed morphological and intracellular structural changes. Indeed, the authors observed the disappearance of cell walls, membrane destruction, and the leakage of intracellular components [40].

Other compounds, like saponins, derived from steroids or triterpenoid glycosides, occur in many plants and affect the microbial cells following the permeabilization of the membrane [2]. As reported by Jing et al., American ginseng saponins and Asian ginseng leaf saponins possessed anti-listeria activity, both in vitro and in mice infected with the pathogens [89].

All parts of plants, and in particular the leaves, steam, and roots, can present tannins [2]. Also, tannins possess antimicrobial activity by inactivating cell proteins (as adhesins, enzymes, and transport proteins) and forming complex proteins [2]. The anti-listeria activity of tannic acid was documented in 2018 by de Almeida Roger et al. [90]. In the same direction, in 2015, Xu et al. demonstrated that tannin-rich fractions from pomegranate rind possess good anti-listeria activity [91]. Procyanidins isolated from laurel wood demonstrated activity against vegetative cells of *L. monocytogenes,* as *Caesalpinia spinosa* (Molina) *Kuntze* is rich in gallotannins and the tannins of *Mangifera indica* seeds. Finally, the proanthocyanidins from grape seeds and *Cinnamomum zeylanicum* have reported anti-listeria activity [92]. Other tannins extracted from chestnut wood, grape, oak gall, and oak trees were able to totally inhibit the growth of *L. monocytogenes* [93]. However, the chemicals found in plants are often in the glycosylated form, which exerts a less extended activity compared to the aglycone form that can be activated via enzymatic hydrolysis [94].

Besides all the compounds that have been mentioned for their defence against pathogens, plants can synthesize proteins as primary metabolites that play a role as enzymes within the plant itself, such as proteinases, amylases, oxydases, etc. [2]. Apart from these enzymes, they can synthesize small peptides with a low molecular weight (about 10 kDa), called antimicrobial peptides. This group includes various peptides such as densins, knottin-like peptides, lipid transfer proteins, heveins, snakin, etc. [2]. For some of these, the anti-listeria activity is well documented. For example, recently, Pachero-Cano et al. studied the anti-listeria activity of defensins from broccoli seeds. In this study, the crude extract of purified defensins were studied, demonstrating their activity [95]. Most of the peptides of this superfamily were isolated from seeds, apart from other parts of plants [35,40]. Like other antimicrobial peptides, defensins preferably exhibit antimicrobial activity through the perturbation of the membrane.

Another group of proteins that possesses anti-listeria activity is called lipid transfer proteins. They are abundant in various plants, e.g., rice and spinach. These proteins play a role in many physiological functions, such as antimicrobial defence, signalling, and intracellular lipid transport [96]. These peptides, isolated from *Chelidonium majus L*. belonging to Papaveraceae and *Triticum turgidum,* have recently demonstrated activity against *Listeria* [96,97]. Instead, potatoes were identified, and some isolated snaking peptides (such as SN-1) also showed activity against *L. monocytogenes* [98,99,100].

## 3. Anti-Listeria Effect of the Extracts Obtained from Fermented Plant-Based Matrices

Comparing the number of works available in the literature (that deal with the fermentation of plant matrices to produce compounds with activity towards *L. monocytogenes*) with those extracting antimicrobial activity from plant matrices, it is immediately apparent that their number is limited (Table 2). However, especially in recent years, is of great interest to search for innovative strategies for the production of compounds with antimicrobial properties that can be employed for food safety, and environmental and economic growth, following the Sustainable Development Goals established in 2015 by the United Nations General Assembly [101]. In this direction, the fermentation of plant matrices could be an innovative and useful technique for the production of antimicrobials that are active against *L. monocytogenes*.

### Compounds Exerting Anti-Listeria Activity Released during the Fermentation Process

During the fermentation process, microorganisms can activate different metabolic pathways for the production/release of inhibitory compounds [120]. From the carbon metabolism, various compounds such as organic acids, phenolics, hydrogen peroxide, acetaldehyde, acetoin, etc., can be liberated, as well as from the nitrogen metabolism (i.e.**,** bacteriocins, peptides, etc.) [120], and can carry out their activities individually or synergically. Different works report the production of various compounds, suggesting that the anti-listeria activity observed after the fermentation process can be related to the synergistic activity of more than one compound [103,105].

Organic acids are one of the main end products produced during the fermentation process. Different are those produced, such as lactic acid, acetic acid, propionic acid, formic acid, succinic acid, citric acid, fumaric acid, malic acid, etc. [121]. Generally, they act on the membrane permeability, changing the cellular pH and inhibiting the enzymatic activity and metabolism [121,122]. For example, when the pH of the environment drops with the presence of lactic acid, the undissociated lactic acid diffuses over the membrane, reducing the cytoplasmatic pH, DNA function, and structural protein expression of *L. monocytogenes* [122,123]. Hwang et al. reported their increase during the fermentation of *P. densiflora* with *Lactiplantibacillus plantarum* and *Saccharomyces cerevisiae,* which is probably related (with other compounds released during the fermentation) to the anti-listeria activity observed in the same work [104]. In addition to organic acids, antimicrobial peptides can also be produced during the fermentation process. For example, Muhialdin et al. studied the fermentation of bitter beans (*Parkia speciosa)* with *Limosilactobacillus fermentum,* observing an increase in anti-listeria activity, attributable to three different peptides released during the fermentation process [108]. The mechanism of action behind the antimicrobial activity of peptides appears to be due to membrane permeabilization through the formation of pores and channels [121,124]. Furthermore, many other compounds can be produced during fermentation, such as hydrogen peroxide, CO_2_, diacetyl, etc., that can cause enzyme inhibition, membrane permeabilization, and instability in the cell wall [121,124]. However, in most of the papers available, the fermentation process seems to affect the composition of the fermented matrices, leading to the release of more than one compound with potential anti-listeria activity. Among these compounds, more than one author reported variation in the phenolic content, such as Kothari et al., who observed that the fermentation of Chinese chive with lactic acid bacteria led to an increase in flavonols such as quercetin, kaempferol, myricetin, rutin, and isorhamnetin, which are compounds exerting antimicrobial activity [125]. Instead, Muhialdin et al. identified many anti-listeria compounds in fermented ginger paste (including butyric acid, lactic acid, acetoin, and citric acid) with epicatechin among them [108]. Spent coffee fermented with *Bacillus clausii* led to an increase in the total phenolic and flavonoid contents, which the authors correlate with the improved activity against *L. monocytogenes* that was observed [113]. Lastly, with the fermentation process, the conversion of glycosylated compounds (often present in plant matrices) into the related aglycon forms can be observed [126,127], reported by Kim et al. during the fermentation of citrus by-products [114].

## 4. Conclusions

This review provided the current understanding of plant extracts as well as fermented plant matrices, and the great reservoir of anti-listeria or potentially anti-listeria compounds that are available in nature. Over the last ten years, plant extracts and fermented plant matrices have been studied for their potential activity against *L. monocytogenes* and their future involvement as preservatives of natural origin. This review encloses interesting findings from the analysis of more than 100 plants or their parts located in various areas of the world, emphasizing the great interest related to this topic. Concurrently, despite the limited literature regarding the fermentation of plant matrices and their antimicrobial activity, the observed results appear to be interesting and promising, even in the face of the Sustainable Development Goals established in 2015 by the United Nations General Assembly. Different compounds observed in plant extracts as well as in fermented matrices seem to be reconnectable with the anti-listeria activity observed; however, in this sense, a multidisciplinary approach could elucidate the compounds affecting the growth of *L. monocytogenes* and their mode of action and could pave the way for new insights combining different antimicrobials to obtain a synergistic or an additive effect against this pathogen, making them more economically favourable.

## Figures and Tables

**Table 1:** Anti-listeria activity of plant extracts.

Matrix	Part of Plant	Solvent Employed	Anti-Listeria Activity	Reference
Published in 2023				
*Nelumbo nucifera*	Leaves	Ethanol	10.6 mm inhibition (10 mg/disk)	[11]
*Cocos nucifera*	Leaves	Ethanol	7.8 mm inhibition (10 mg/disk)	[11]
*Nypa fruticans*	Leaves	Ethanol	8.9 mm inhibition (10 mg/disk)	[11]
*Nepenthes mirabilis*	Leaves	Ethanol	10.9 mm inhibition (10 mg/disk)	[11]
*Crocus sativus* L.	Flower by-product	Diethyl ether	MIC 25 mg/mL MBC 50 mg/mL	[12]
*Crocus sativus* L.	Flower by-product	Ethyl acetate	MIC 50 mg/mL MBC > 100 mg/mL	[12]
Propolis		Ethanol	11–30 mm inhibition zone (20 μL)	[13]
*Alchornea trewioides*	Leaves and branch	Ethanol/Distilled water	MIC 6.2 mg/mL	[14]
*Erodium stephanianum*	Leaves and branch	Ethanol/Distilled water	MIC 25 mg/mL	[14]
*Gentiana lutea*	Leaves	Water/Ethanol	MIC 10 mg/mL	[15]
*Gentiana lutea*	Root	Water/Ethanol	MIC 10 mg/mL	[15]
*Prangos ferulacea*	Plant	Distilled water	MIC 16 mg/mL MBC 128 mg/mL	[16]
Liverwort *F. dilatata*	Plant	Ethanol	MIC 0.26 mg/mL	[17]
Liverwort *F. dilatata*	Plant	Water	MIC 21.44 mg/mL	[17]
*Dodonaea angustifolia* (L.f.)	Leaves	Methanol	9.7 mm inhibition (100 μL at 200 mg/mL)	[18]
*Dodonaea angustifolia* (L.f.)	Flowers	Methanol	9.3 mm inhibition (100 μL at 200 mg/mL)	[18]
*Eugenia uniflora* L. (Pitangueira)	Leaves	Water/Ethanol	MIC 12.5 mg/mL	[19]
*Origanum vulgare* L.	Leaves	Water	MIC 135 μg/mL	[20]
*Origanum dictamnus* L.	Leaves	Water	MIC 80 μg/mL	[20]
*Hypericum perforatum* L.	Leaves	Water	MIC 30 μg/mL	[20]
*Origanum majorana* L.	Leaves	Water	MIC 5 μg/mL	[20]
*Mentha spicata* L.	Leaves	Water	MIC 5 μg/mL	[20]
*Annona muricata* (soursop)	Leaves	Ethanol	MIC 50–100 mg/mL MBC 100–200 mg/mL	[21]
*Morinda citrifolia* (Noni)	Leaves	Ethanol	MIC 25–50 mg/mL MBC 50–100 mg/mL	[21]
*Barringtonia acutangula* (Jik)	Leaves	Ethanol	MIC 25–50 mg/mL MBC 50–100 mg/mL	[21]
*Berberis libanotica* Ehrenb.	Leaves	Dichloromethane	MIC 39 μg/mL	[22]
*Berberis libanotica* Ehrenb.	Fruit	Methanol	MIC 625 μg/mL	[22]
*Berberis libanotica* Ehrenb.	Fruit	Cyclohexane	MIC 4.8 μg/mL	[22]
*Berberis libanotica* Ehrenb.	Fruit	Ethylacetate	MIC 78–312 μg/mL	[22]
*Justicia Pectoralis Jacq.* Chambá	Leaves	Water	MIC 13 mg/mL MBC 18 mg/mL	[23]
*Justicia Pectoralis Jacq.* Chambá	Leaves	Water/Ethanol	MIC 25–35 mg/mL MBC 35–100 mg/mL	[23]
*Azadirachta indica* L. (Neem)	Leaves	Methanol	11–12 mm inhibition zone (50 μL at 50 μg/mL)	[24]
*Melia azedarach* L. (China tree)	Leaves	Methanol	9–11 mm inhibition zone (50 μL at 50 μg/mL)	[24]
*P. atlantica*	Leaf buds	Methanol/Water	MIC 39 μg/mL MBC 1250 μg/mL	[25]
Red onion	Peel	Water/Ethanol	12.9 < MIC < 25.8 mg QdGE/g	[26]
Published in 2022				
*Rubus fruticosus*	Leaves	Water/Ethanol	MIC 2 mg/mL	[27]
*Juniperus oxycedrus*	Needles	Water/Ethanol	MIC 2 mg/mL	[27]
*Rosa damascena*	Flowers	Water/Ethanol	MIC 20.8 mg/mL MBC 41.7 mg/mL	[28]
*Pistacia lentiscus*	Leaves	Ethanol	MIC 0.04 mg/mL MBC 3.84 mg/mL	[29]
*Rosmarinus officinalis*	Leaves	Ethanol	MIC 3.84 mg/mL MBC 3.84 mg/mL	[29]
*Erica multiflora*	Leaves	Ethanol	MIC 3.84 mg/mL MBC 3.84 mg/mL	[29]
*Calicotome villosa*	Leaves	Ethanol	MIC 3.84 mg/mL MBC 12 mg/mL	[29]
*Phillyrea latifolia*	Leaves	Ethanol	MIC 3.84 mg/mL MBC 12 mg/mL	[29]
*Crocus sativus* L.	Petals	Ethanol/Water	MIC 4.33 mg/mL MBC 17.35 mg/mL	[30]
*Anacylus clavatus*	Flowers	Ethanol	MIC 41.66 mg/mL MBC 166.66 mg/mL	[31]
*Allium ursinum*	Leaf	Water	MIC 28 mg/mL MBC 29 mg/mL	[32]
*Juglans regia* L.	Flower	Methanol	MIC 0.63 mg/mL MBC 2.5 mg/mL	[33]
*Punica granatum* L. (Pomegranate)	Peel	Water	MIC 19 mg/mL	[34]
*Punica granatum* L. (Pomegranate)	Peel	Ethanol	MIC 24 mg/mL	[34]
*M. officinalis*	Dry plant	Ethanol/Water	MIC 1 mg/mL MBC 2 mg/mL	[35]
*O. vulgare*	Dry plant	Ethanol/Water	MIC 1 mg/mL MBC 2 mg/mL	[35]
*M. chamomilla*	Dry plant	Ethanol/Water	MIC 0.5 mg/mL MBC 1 mg/mL	[35]
*T. vulgare*	Dry plant	Ethanol/Water	MIC 0.5 mg/mL MBC 1 mg/mL	[35]
*O. basilicum*	Dry plant	Ethanol/Water	MIC 1 mg/mL MBC 2 mg/mL	[35]
*S. officinalis*	Dry plant	Ethanol/Water	MIC 0.5 mg/mL MBC 1 mg/mL	[35]
*Origanum compactum*	Aerial Parts	Water/Ethanol	MIC 41 mg/mL MBC 83 mg/mL	[36]
*Thymus vulgaris* L.	Whole plant	Ethanol	MIC 3.1–50.0%	[37]
*Thymus vulgaris* L.	Whole plant	Cold water	MIC > 50%	[37]
*Thymus vulgaris* L.	Whole plant	Hot water	MIC > 50%	[37]
*Thymus vulgaris* L.	Whole plant	Acetone	MIC 6.3–50%	[37]
*Thymus vulgaris* L.	Seeds	Ethanol	MIC 3.1–25%	[37]
*Thymus vulgaris* L.	Seeds	Cold water	MIC > 50%	[37]
*Thymus vulgaris* L.	Seeds	Hot water	MIC > 50%	[37]
*Thymus vulgaris* L.	Seeds	Acetone	MIC 12.5–50%	[37]
*Thymus vulgaris* L.	Leaves	Ethanol	MIC 3.1–25%	[37]
*Thymus vulgaris* L.	Leaves	Cold water	MIC > 50%	[37]
*Thymus vulgaris* L.	Leaves	Hot water	MIC > 50%	[37]
*Thymus vulgaris* L.	Leaves	Acetone	MIC 3.1–50%	[37]
*Thymus vulgaris* L.	Stems	Ethanol	MIC 6.3–50%	[37]
*Thymus vulgaris* L.	Stems	Cold water	MIC > 50%	[37]
*Thymus vulgaris* L.	Stems	Hot water	MIC > 50%	[37]
*Thymus vulgaris* L.	Stems	Acetone	MIC 12.5–50%	[37]
Negro pepper		Ethanol	0.1% < MIC < 0.2% MBC 0.2%–MBC > 0.4%	[38]
Negro pepper		DMSO	0.05% < MIC < 0.2% 0.1% < MBC < 0.4%	[38]
Negro pepper		Methanol	0.1% < MIC < 0.4% 0.2% < MBC < 0.4%	[38]
Negro pepper		Water	MIC > 0.4% MBC > 0.4%	[38]
Clove		Ethanol	0.2% < MIC < 0.4% MBC ≥ 0.4%	[38]
Clove		DMSO	0.2% < MIC < 0.4% MBC 0.4%	[38]
Clove		Methanol	MIC 0.2% 0.2% < MBC < 0.4%	[38]
Clove		Water	MIC > 0.4% MBC > 0.4%	[38]
Broccoli	Seeds	Methanol/Water	MIC 0.8 mg/mL	[39]
*Corydalis turschaninovii*	Rhizome	Ethanol/Water	MIC 3.12 mg/mL MBC 6.25 mg/mL	[40]
Published in 2021				
*Maclura pomifera* (Osage orange)	Leaves	Ethanol	MIC 10–30 mg/mL	[41]
*Rhus tripartita*	Leaves	Aceton	MIC 500 μg/mL MBC 500 μg/mL	[42]
*Ziziphus lotus*	Leaves	Aceton	MIC 500 μg/mL MBC 2000 μg/mL	[42]
*Origanum ehrenbergii* Boiss	Aerial part	Cyclohexane	MIC 313 μg/mL	[43]
*Origanum ehrenbergii* Boiss	Aerial part	Dichloromethane	4 < MIC < 19 μg/mL	[43]
*Satureja kitaibelii* Wierzb.	Aboveground flowering parts	Water	MIC 2.08 mg/mL	[44]
*Crocus sativum Linn*	Flower stamens	Diethyl ether	MIC 9 mg/mL MBC 9 mg/mL	[45]
*Rosa gallica* var. aegyptiaca	Leaves	Methanol	16 mm inhibition zone (40 µL at 10 mg/40 µL)	[46]
*Rosa gallica* var. aegyptiaca	Leaves	Water/Methanol	17 mm inhibition zone (40 µL at 10 mg/40 µL)	[46]
*Rosa gallica* var. aegyptiaca	Leaves	Water	12 mm inhibition zone (40 µL at 10 mg/40 µL)	[46]
*Humiria balsamifera* (Aubl.)	Leaf	Ethyl acetate	MIC 3.12 mg/mL	[47]
*Humiria balsamifera* (Aubl.)	Leaf	Methanol	MIC 3.12 mg/mL	[47]
Wild thyme	Plant	Water/Ethanol	MIC 0.63 mg/mL MBC 2.5 mg/mL	[48]
*Punica granatum L.*	Pulp	Ethanol	10.0 mm inhibition zone (10 μL at 50 mg/mL)	[49]
*Punica granatum L.*	Peel	Ethanol	14.0 mm inhibition zone (10 μL at 50 mg/mL)	[49]
*Garlic*	Bulb	Water	MIC 8–32 μg/mL	[50]
*Onion*	Bulb	Water	MIC 4–32 μg/mL	[50]
Published in 2020				
*Bouea macrophylla*	Leaves	Ethanol	17.83–16.16–14.83–13.5–11.50 mm inhibition zone (100 µL at 500, 100, 10, 1, 0.1 mg/mL)	[51]
Winter savoury	Leaves	Water/Ethanol	MIC 20 mg/mL MBC 20 mg/mL	[52]
*Nephelium lappaceum L.* (Rambutan)	Fruit peel	Methanol	Growth inhibition at 1000, 100, and 10 μg GAE/mL	[53]
*Camellia sinensis* (L.) O. Kuntze	Non-fermented leaves and buds	Distilled water	MIC 1.25 mg/mL	[54]
*Curcuma longa L.*	Rhizomes	Distilled water	MIC 1.25 mg/mL	[54]
*Loranthus europaeus*	Berry	Sodium Acetate/DTT/PMSF	MIC 0.28 mg/mL MBC 0.38 mg/mL	[55]
*Syzygium cumini* (L.) Skeel	Pulp	Ethanol/Methanol/Acetone	MIC > 0.78 mg GAE/g pulp	[56]
*Syzygium cumini* (L.) Skeel	Seed	Ethanol/Methanol/Acetone	MIC 5.65 mg GAE/g pulp	[56]
*Ziziphus lotus*	Leaf	Methanol	10.0–12.0 mm inhibition (20 μL at 10 mg/mL)	[57]
*Ziziphus mauritiana*	Leaf	Methanol	12.0 mm (20 μL at 10 mg/mL)	[57]
*Persea americana* Mill (Avocado)	Peel	Ethanol/Water	MIC ≥ 0.75 mg/mL	[58]
*Anacardium occidentale* L. (Cashew apple)	Residual fibres	Water	13.0 and 11.0 mm inhibition zone (50 μL at 100, 50 mg/mL)	[59]
*Hibiscus sabdariffa* L.	Flower and beefsteak	Acidified water/Methanol/Acetone	MIC 200 mg/L of GAE MBC 400 mg/L of GAE	[60]
*Trichilia emetica*	Leaves	Methanol	MIC 10 mg/mL	[61]
*Passiflora foetida*	Whole plant	Methanol	MIC 5 mg/mL	[61]
*Salvia nemorosa*	Whole plant	Methanol	MIC 5 mg/mL	[61]
*Sambucus ebulus*	Whole plant	Methanol	MIC 10 mg/mL	[61]
*Baphia racemosa*	Root	Methanol	MIC 2.5 mg/mL	[61]
*Sansevieria hyacinthoides*	Root	Methanol	MIC 2.5 mg/mL	[61]
*Desmodium adscendens*	Whole plant	Methanol	MIC 5 mg/mL	[61]
*Eriosema preptum*	Whole plant	Methanol	MIC 10 mg/mL	[61]
*Darlingtonia californica*	Leaves	Methanol	MIC 10 mg/mL	[61]
*Proboscidea louisianica*	Seed pod	Methanol	MIC 10 mg/mL	[61]
*Alnus barbata*	Leaves and twigs	Methanol	MIC 5 mg/mL	[61]
*Botrychium multifidum*	Root	Methanol	MIC 5 mg/mL	[61]
*Cudrania tricuspidata*	Leaves	Ethanol/Water	16, 19, 24 and 24 mm inhibition zone (80 μL at 1%, 2.5%, 5.0% and 10%)	[62]
Cranberry	Pomace	Ethanol	MIC 2–4 mg/mL	[63]
Published in 2019				
Noni	Fruit	Ethanol/Water	15.61–18.75–20.26–22.43 mm inhibition zone (24, 40, 56, and 80 mg/disc)	[64]
*Moringa stenopetala*	Leaves	Ethanol	MIC 500 μg/mL	[65]
*Moringa stenopetala*	Leaves	Methanol	MIC 250 μg/mL	[65]
*Moringa stenopetala*	Leaves	Chloroform	MIC 125 μg/mL	[65]
*Moringa stenopetala*	Leaves	Water	MIC 250 μg/mL	[65]
*Punica granatum* (pomegranate)	Peels	Water	7.82 < MIC < 31.25 mg/mL	[66]
*Vitis vinifera x (Vitis labrusca x Vitis riparia)* (Black grape)	Residues	Carbohydrase treatment—Ethanol/Water	50 < MIC < 100 mg/mL	[67]
*Malus domestica cv. Jonagold* (Apple)	Residues	Carbohydrase treatment—Ethanol/Water	MIC 50 mg/mL or MIC> 100 mg/mL	[67]
*Hylocereus megalanthus* (Yellow pitahaya)	Residues	Carbohydrase treatment—Ethanol/Water	MIC ≥ 100 mg/mL	[67]
*Eucalyptus camaldulensis*	Leaves	Ethanol	MIC 64–128 μg/mL MBC 265–512 μg/mL	[68]
Cranberry	Pomace	Ethanol	100% growth inhibition (at 6.6 and 3.3%)	[69]
Cranberry	Pomace	Water	100% growth inhibition (at 6.6 and 3.3%)	[69]
*Psoralea corylifolia*	Seeds	Ethanol	MIC 50 μg/mL MBC 100 μg/mL	[70]
Published in 2018				
*Echium arenarium* (Guss.)	Aerial parts	Ethanol–Ethyl acetate	18.0 mm inhibition (1 mg) MIC > 1 mg/mL	[71]
*Ajuga iva* (L.)	Aerial part	Methanol	3 mm inhibition zone (25 μL at 50 mg/mL)	[72]
*Ajuga iva* (L.)	Aerial part	Water	6.6 < MIQ <1.3 mg/disk	[72]
*Alpinia galanga* (Linn.) Swartz. (Greater galangal)	Flowers	Methanol/Water	1.15 < MIC < 12.3 mg/mL	[73]
*Alpinia galanga* (Linn.) Swartz. (Greater galangal)	Flowers	Methanol	0.02 < MIC < 0.03 mg/mL	[73]
Published in 2017				
Olive	Leaf	Ethanol/Water	MIC 62.6 mg/mL	[74]
*Ugni molinae* Turcz.	Fruit	Ethanol	MIC 0.6 mg/mL MBC 0.9 mg/mL	[75]
*Ugni molinae* Turcz.	Fruit	Ethanol/water	MIC 0.2 mg/mL MBC 0.5 mg/mL	[75]
*Ugni molinae* Turcz.	Fruit	Water	MIC 1.8 mg/mL MBC 2.2 mg/mL	[75]
*Ugni molinae* Turcz.	Leaf	Ethanol	MIC 0.2 mg/mL MBC 0.5 mg/mL	[75]
*Ugni molinae* Turcz.	Leaf	Ethanol/Water	MIC 0.07 mg/mL MBC 0.09 mg/mL	[75]
*Ugni molinae* Turcz.	Leaf	Water	MIC 0.7 mg/mL MBC 0.9 mg/mL	[75]
*R. tingitanus*	Leaves	Ethanol/Water	MICs 5–0.625 mg/mL	[76]
Grapes *(V. vinifera L.)* var. Red Globe	Stem	Ethanol/Water	MIC 18 mg/mL MBC > 24 mg/mL	[77]
*Cinnamon javanicum*	Plant	Acetone/Methanol/Water	0.13 < MIC < 8 mg/mL	[78]
*Adhatoda vasica*	Leaves	Ethanol	MIC 100 mg/mL	[79]
Published before 2017				
*Vitis vinifera L.*	Seeds	Hexane	MIC 3.12 mg/mL MBC 6.25 mg/mL	[80]
*Vitis vinifera L.*	Seeds	Dichloromethane	MIC 3.12 mg/mL MBC 12.5 mg/mL	[80]
*Vitis vinifera L.*	Seeds	Ethyl acetate	MIC 3.12 mg/mL MBC 6.25 mg/mL	[80]
*Vitis vinifera L.*	Seeds	Acetone	MIC 6.25 mg/mL MBC 12.5 mg/mL	[80]
*Vitis vinifera L.*	Seeds	Ethanol	MIC 1.56 mg/mL MBC 12.5 mg/mL	[80]
*Vitis vinifera L.*	Seeds	Water	MIC 12.5 mg/mL MBC 25 mg/mL	[80]
*Rhodomyrtus tomentosa*	Leaves	Ethanol/Water	MIC 16–32 μg/mL MBC 128–512 μg/mL	[81]
*Coffea arabica* (Roasted coffee)	Spent coffee	Water	MIC 20 mg/mL	[82]
*Coffea arabica* (Roasted coffee)	Coffee brew	Water	MIC 30 mg/mL	[82]
*Coffea canephora* var. robusta (Roasted coffee)	Spent coffee	Water	MIC 20 mg/mL	[82]
*Coffea canephora* var. robusta (Roasted coffee)	Coffee brew	Water	MIC 16.3 mg/mL	[82]
*Artocarpus heterophyllus* L.	Shell	Acetone/Water	MIC 4.2 mg/mL	[83]
*Artocarpus heterophyllus* L.	Shell	Methanol/Water	MIC 4.2 mg/mL	[83]
*Artocarpus heterophyllus* L.	Shell	Ethanol/Hexane/Water	MIC 4.2 mg/mL	[83]
*G.biloba*	Seed coat	Chloroform	MIC 8.3–16.6 μg/mL MBC 16.6–33.3 μg/mL	[84]
*Veronica montana* L.	Aerial parts	Water	MIC 7.5 mg/mL MBC 15.0 mg/mL	[85]

MIC: minimum inhibitory concentration; MBC: minimum bactericidal concentration; MIQ: minimum inhibitory quantity (μg/disk); mg QdGE/g: mg quercetin-3, 4′-diglucoside equivalent per gram.

**Table 2:** Anti-listeria activity of fermented plant-based matrices.

Matrix	Part of Plant	Microorganism Employed in Fermentation	Type of Fermented Product	Anti-Listeria Activity	Reference
Published in 2023					
*Ulmus davidiana var. japonica* (Ulmaceae)	Root bark	*Bacillus licheniformis*	Extract	Fermented: MIC 75 mg/mL MBC 125 mg/mL Unfermented: MIC 100 mg/mL MBC 150 mg/mL	[102]
Thyme (*Thymus vulgaris*), lemon verbena (*Lippia citriodora*), rosemary (*Rosmarinus officinalis*), fennel (*Foeniculum vulgare*), and peppermint (*Mentha piperita*)	Leaves	Symbiotic culture between yeast and acetic acid bacteria	Entire product	Inhibition of *L. monocytogenes,* activity (acidity is important for antibacterial activity)	[103]
*P. densiflora*	Pine needles	*Lpb. plantarum*, *Saccaromices cerevisiae* and co-culture	Entire product	*Lpb. plantarum:* 10–13 mm inhibition zone (100 μL) *S. cerevisiae:* 9 mm inhibition zone (100 μL) Co-culture: 10–12 mm inhibition zone (100 μL)	[104]
Published in 2021					
Black tea	Leaves	Symbiotic culture between yeast and acetic acid bacteria	Entire product	15 mm inhibition zone (100 μL)	[105]
Tomato	Peels and seeds	*Lacticaseibacillus rhamnosus*	Extract	MBC 12.5–100 mg/mL	[106]
Melon	Fruits	*L. rhamnosus*	Extract	MBC 12.5–25 mg/mL	[106]
Carrot	Tuber	*L. rhamnosus*	Extract	MBC 6.25–MBC > 50 mg/mL	[106]
Published in 2020					
Green Tea	Leaves	SCOBY for Kombucha	Entire product	MIC 250 μL/mL	[107]
Black Tea	Leaves	SCOBY for Kombucha	Entire product	MIC 250 μL/mL	[107]
*Parkia speciosa* (bitter beans)	Seeds	*Limosilactobacillus fermentum*	Extract	57% growth inhibition	[108]
*Zingiber officinale* (ginger)	Rhizome	Spontaneous fermentation	Diluted ginger paste	97.6% growth inhibition	[109]
Curly kale	Juice	Spontaneous fermentation	Juice	Ca. 50% growth inhibition	[110]
*Salvia miltiorrhiza* (red sage)	Roots	*Aspergillus oryzae*	Extract	MIC 1 mg/mL (against 2 mg/mL of unfermented sample)	[111]
Published in 2019					
Tomato	Peels and seeds	*Lpb. plantarum, Lacticaseibacillus casei, Lacticaseibacillus paracasei and L. rhamnosus*	Extract	Ca. 14–16 mm inhibition zone (40%)	[112]
Melon	Fruits	*Lpb. plantarum, L. casei, L. paracasei and L. rhamnosus*	Extract	Ca. 12–16 mm inhibition zone (60%)	[112]
Carrot	Tuber	*Lpb. plantarum, L. casei, L. paracasei and L. rhamnosus*	Extract	Ca. 2–12 mm inhibition zone (60%)	[112]
Published in 2018					
Coffee	Spent ground	*Bacillus clausii*	Extract	MIC 10 mg/mL (against 30 mg/mL unfermented sample)	[113]
Published in 2017					
*Citrus unshiu*	Flesh byproducts	Nuruk (*Aspergillus* sp., *Rhizopus* sp., *Saccharomyces cerevisiae, Bacillus subtilis*, and lactic acid bacteria)	Extract	9 mm inhibition zone (20 μL at 100 mg/mL)	[114]
*Citrus unshiu*	Peel byproducts	Nuruk (*Aspergillus* sp., *Rhizopus* sp., *Saccharomyces cerevisiae, Bacillus subtilis*, and lactic acid bacteria)	Extract	9 mm inhibition zone (20 μL at 100 mg/mL)	[114]
*Allium sativum L*	Bulb	*Lpb. plantarum*	Extract	Ca. 5–9 mm inhibition zone (100 μL at 300 mg/mL)	[115]
Published before 2017					
*Allium tuberosum*	Plant	*Leuconostoc mesenteroides*	Entire product	Ca. 13 mm inhibition zone (100 μL)	[116]
*Aloe vera*	Leaves	*Lpb. plantarum*	Fermented supernatant	20 mm inhibition zone (200 μL)	[117]
Polished rice		Spontaneous fermentation	Entire product	12–13 mm inhibition zone (50 μL)	[118]
*Melissa* *officinalis* L.	Arial parts	SCOBY (Consortium of *Saccharomycodes* *ludwigii*, *S. cerevisiae*, *Saccharomyces bisporus*, *Torulopsis* sp. and *Zygosaccharomyces* sp.) and two bacterial strains of the *Acetobacter* genus)	Entire product	11–17 mm inhibition zone (100 μL)	[119]
